# Subgross Morphology, the Sick Lobe Hypothesis, and the Success of Breast Conservation

**DOI:** 10.4061/2011/634021

**Published:** 2011-05-05

**Authors:** Tibor Tot

**Affiliations:** Department of Pathology and Clinical Cytology, Central Hospital Falun, 79182 Falun, Sweden

## Abstract

Breast carcinoma has a complex subgross morphology in the majority of cases. The malignant transformation usually involves a single breast lobe and may demonstrate peripheral, segmental, or lobar growth patterns in the in situ phase. During the invasive phase, the tumor may grow beyond the borders of the affected lobe. The dimensions of the involved lobe and the pattern of its involvement determine the extent of the disease in the early phase, with the size, type, and position of the invasive foci being additional determinants in more advanced cases. Breast carcinomas of limited extent (occupying a tissue area <40 mm) are proper candidates for breast-conserving surgery. In other cases, careful individual preoperative assessment of disease extent is necessary in making decisions about the most appropriate surgical approach, taking into account the position of the lesion(s) within the breast, the dimensions of the breast, and patient preference.

## 1. Introduction

Breast-conserving surgery completed with postoperative irradiation results in good local control of the disease, with relatively few ipsilateral local recurrences [1, 2]. A considerable number of patients, however, still experience local recurrence, even in some of the cases when the surgical margins of the resection have been judged to be cancer free. This number is much higher if postoperative irradiation is omitted [1, 2]. In addition to the possibility of erroneous assessment of the surgical margins as an explanation, another possibility is the leaving behind of foci of cancer or risk tissue within the breast after seemingly complete surgery [3].

Most breast carcinomas have a complex morphology that is often evident already at an early stage of the disease [3–8]. Early lesions are often nonpalpable, small, and hardly visible to the naked eye. Nevertheless, despite their small size, early breast carcinomas are often multifocal and extensive [4, 8, 9], and the small individual foci may be spread over an area of several centimeters in volume, resulting in a large extent of the disease. These seemingly contradictory facts indicate the need for using special nonfragmenting histology techniques in all such cases and also emphasize the paramount importance of a detailed radiological-pathological correlation in diagnosing breast carcinoma in the modern era. 

Factors influencing the success of breast-conserving therapy are numerous, with the final determinants of treatment choice being the extent of the disease, ability to tolerate radiotherapy, and patient preference [10]. In this paper, the subgross morphology of breast cancer is discussed in relation to the success of local control of the disease with a special focus on disease extent.

## 2. Theoretical Background

 Breast is a glandular organ with lobar morphology. A breast lobe comprises a single lactiferous duct opening on the nipple, its segmental, subsegmental, and terminal branches with the terminal ductal-lobular units (TDLUs) at the end of the branching tree. The reported number of lobes within a mature breast varies considerably in the literature, 27 being the median in one detailed study [11]. The lobes are individual units with no anastomotic connections between them. 

**Figure 1:**
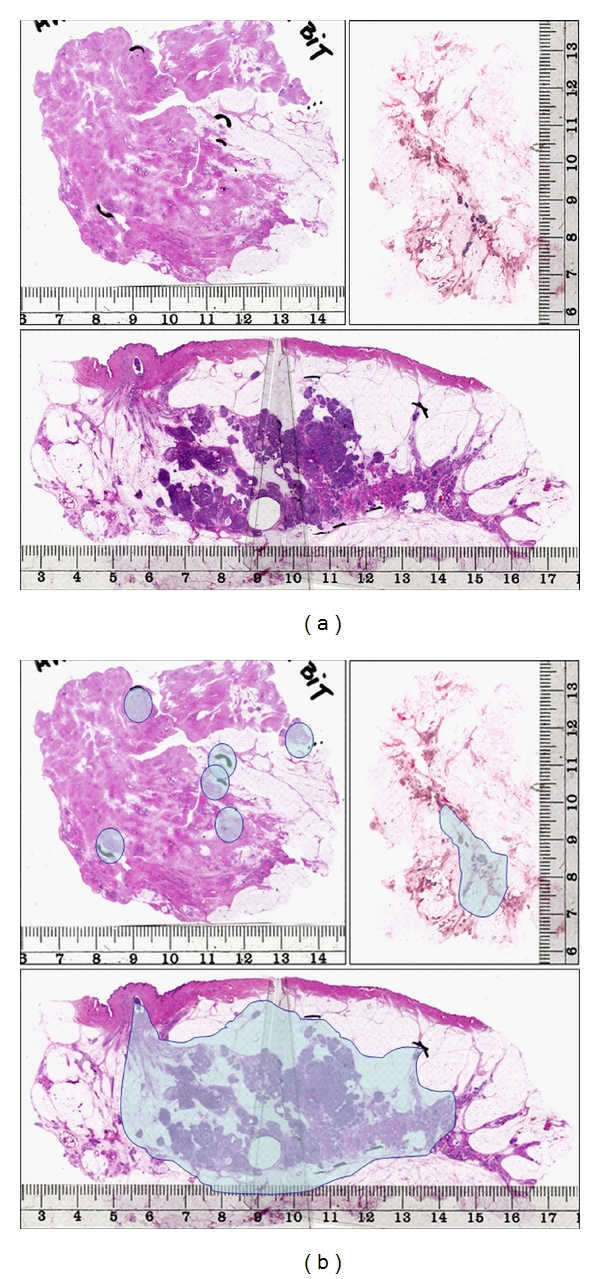
The three basic growth patterns of in situ carcinoma within the sick breast lobe. Upper left: the peripheral pattern; upper right: the segmental pattern; lower image: the lobar pattern. The structures involved by in situ carcinoma, corresponding to the extent of the disease, are marked in the series of images on the right-hand side.

 According to our hypothesis of the sick lobe [12–14], breast carcinoma is a lobar disease in that the simultaneously or asynchronously appearing in situ or invasive tumor foci develop within a single sick lobe and the cancerous structures are confined to the area of the sick lobe at the early stage of the disease. The sick lobe probably contains more or more-sensitive committed progenitor cells than the other lobes of the same breast and is more sensitive to endogenous or exogenous oncogenic stimuli. This hypothesis is congruent with the concept of committed progenitor cells [15], as well as with the concept of mammary field cancerization [16]. The most important implication of these concepts is that an area several centimeters in size of genetically altered tissue may exist in the breast and breast cancer develops within this area rather than at one single point. According to our related hypothesis, the theory of biological timing [9, 13], the time of complete malignant transformation of the committed progenitor cells, is determined by the number of required additional genetic alterations, which are mostly acquired during the division of these cells. This transformation may appear in a single locus within the sick lobe, at more than one locus at the same time or with a considerable time difference, or at a large number of loci leading to a unifocal, multifocal, or diffuse malignant process, respectively. Although the variations in breast cancer morphology are practically unlimited, three patterns of cancer development seem to be the most typical at the early stage: the peripheral pattern (involving the TDLUs), the segmental pattern (involving a segmental duct together with its branches and terminal units), and the lobar pattern (involving the entire sick lobe or large parts of it) [14].  Figure 1 illustrates these patterns for in situ carcinoma.

As demonstrated in numerous studies [17–19], further mutations in the malignant cells and the cells of the surrounding stroma may result in cancer cells losing their ability to maintain the myoepithelial layer and the basal membrane around the ducts and terminal units, and the normal periductal, intralobular, and interlobular stroma undergoes remodeling. Both individual cancer cells and groups of such cells may come into direct contact with stromal elements and be entrapped in the remodeled stroma. They may also come into contact with the prelymphatic spaces and lymphatic vessels, invade them, and be transported via the lymphatic spaces and lymphatic system within (or outside) the breast. In this way, the invasive tumor may spread beyond the area of the sick lobe. Through proliferation of the malignant cells, the invasive component of the tumor may grow, not only around the pre-existing in situ process but also, following its intramammary spread, at distant sites. The tumor foci may eventually coalesce, giving a larger tumor mass with more complex morphology. By further mutations and dedifferentiation, new cell clones may appear in the invasive foci, leading to intratumoral and intertumoral heterogeneity within the same breast. Via these mechanisms, the tumor gradually enters the advanced phase.

## 3. Assessing the Subgross Morphology of Early Breast Cancer

In our approach, the distributions of the invasive and in situ components of the same cancer are determined separately. In situ carcinomas are regarded as “unifocal” if they appear to involve a single TDLU or several neighboring TDLUs together with the belonging subsegmental or segmental duct(s). They are designated as “multifocal” if they involve several distant TDLUs with uninvolved breast tissue in between and as “diffuse” if they involve mainly the larger ducts [6–9]. The unifocal pattern of in situ carcinomas corresponds to the segmental pattern, the multifocal pattern to the peripheral pattern, and the diffuse to the lobar pattern of cancer development, as discussed above and as illustrated in Figure 1. These alternative terms reflect two approaches: the peripheral-segmental-lobar designations reflecting the biological approach based on the sick lobe hypothesis and the unifocal-multifocal-diffuse designations reflecting the practical routine diagnostic approach. 

**Figure 2:**
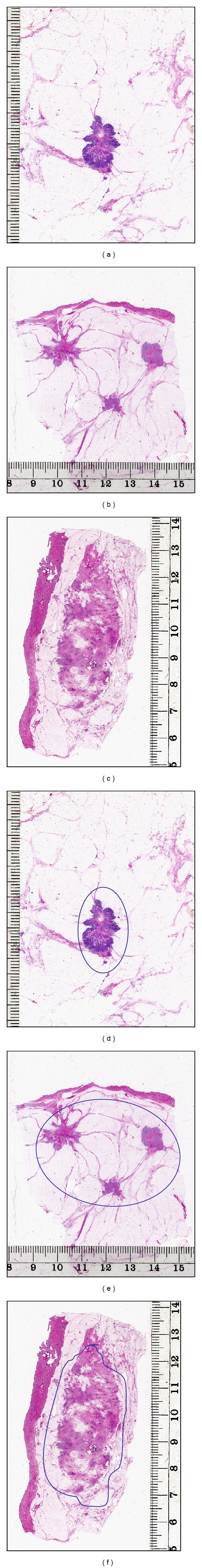
The three basic growth patterns of invasive breast carcinoma. Upper left: unifocal; upper central: multifocal; upper right: diffuse growth pattern. The extent of the disease is marked in the lower series of images.

Invasive lesions are considered to be “unifocal” if only one invasive focus is observed, which may or may not contain an in situ component. “Multifocal” invasive lesions are characterized by the presence of multiple, well-delineated invasive tumor foci separated from each other by uninvolved breast tissue, regardless of the distance between the foci. Tumors dispersed over a large area in the section, much like a spider web, with no distinct tumor mass are classified as “diffuse,” but they are usually large and are not represented among early cancers (Figure 2). Cancers may lack an in situ or an invasive component although most of them have both; any further combination of the in situ and invasive components may characterize an individual case. Theoretically, there are 16 such combinations [6]. In our practical approach, however, after the initial separate assessment of the distribution of the in situ and invasive foci, we combine the findings so that diffuse distribution of the in situ or the invasive component qualifies the lesion as a tumor having a diffuse combined lesion distribution. Multifocality of either the in situ or invasive tumor component, or both, results in multifocal combined lesion distribution. 

**Figure 3:**
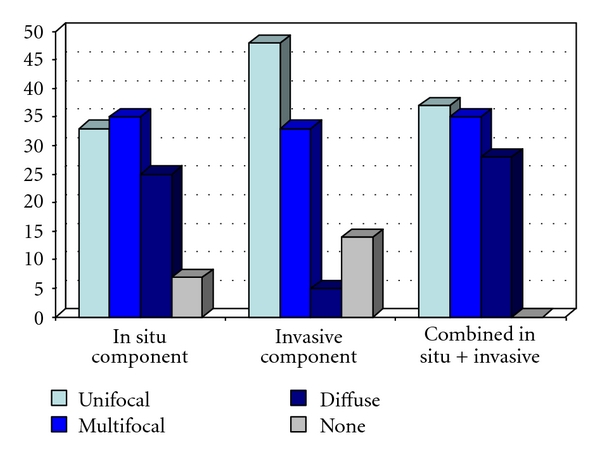
Percentages of carcinomas with unifocal, multifocal, and diffuse subgross patterns regarding the in situ component of the tumor, the invasive component, or both combined. Falun 2008–2010.

Figure 3 demonstrates the percentages of different subgross tumor distribution (growth patterns) regarding the in situ component, the invasive component, and the combined patterns, respectively. The material comprises 565 consecutive cases newly diagnosed at our department, all documented in large-format histology slides. Forty tumors (7%, 40/565) lacked an in situ component, while 80/565 (14%) were purely in situ lesions lacking an invasive component. A total of 25% (138/565) of in situ tumors were diffuse (involving large parts of the ductal system of the sick lobe), but only approximately 5% (26/565) of the tumors showed the typical spider web-like diffuse pattern of the invasive component. The in situ component was unifocal in 33% (189/565) and multifocal in 35% (198/565), while the invasive component was unifocal in half of the cases (48%, 274/565) and multifocal in one third of cases (33%, 185/565). The combined distribution of the in situ and the invasive components was as follows: unifocal in 37% (209/565), multifocal in 35% (198/35), and diffuse in 28% (158/565). 

Thus, the subgross distribution of the lesion is complex in the majority of breast carcinomas, and for its proper assessment, a close and detailed radiologic-pathologic correlation is as important as using adequate nonfragmenting histology techniques. Assessment of lesion distribution is essential because it represents independent morphologic prognostic parameters in breast carcinoma, which are as important as tumor size. Specifically, multifocal and diffuse distribution of the invasive lesions is associated with an increased propensity for metastatic tumor spreading [6–8, 20, 21] and with shortened breast cancer-specific survival [22–25].

## 4. The Extent of the Disease

While lesion distribution is often complex, disease extent is a morphologic parameter that is easier to communicate within the breast team. This parameter is defined as the area or volume of the breast tissue containing all the in situ, invasive, and intravascular malignant tumor foci. Of importance, disease extent and tumor size (defined as the largest dimension of the largest invasive tumor focus within the breast) differ from each other in the vast majority of cases, being equal only in cases of unifocal invasive carcinomas having no in situ component outside the invasive focus, which comprise no more than 15% of our cases.

Breast morphology as perceived in a histology specimen reflects the status of the balance between dynamic progressive and regressive processes that were stopped at the moment of tissue fixation; it is a still frame from an ongoing process. Microscopic analysis of the specimen gives us only limited information about these processes but represents an important checkpoint in the attempt to reconstruct the natural history of a lesion. This reconstruction is particularly valid for determining disease extent. 

It has to be underlined that the extent of the disease is a term relating not to a single component of the tumor, but to all malignant structures within the same breast. The parameter of extensive intraductal component (EIC) [26] is not identical to disease extent. Using our approach, we visualize malignant transformation of the large parts of the ductal tree and/or the lobules within the sick lobe leading to extensive disease in a considerable number of cases. This situation represents a negative prognostic parameter [27], similarly but not identically to that evidenced in cases with EIC [26, 28]. 

### 4.1. Extent of the Disease: The Dimensions of the Involved Breast Lobe

The dimensions of the breast lobes vary considerably within the same breast and also individually. The largest lobe demonstrated in one of the very few related studies comprised 25% of the breast volume, the smallest only 1% of the breast volume [29]. Lobes are larger in the upper outer quadrant of the breast than in the medial parts [30]. In addition, the dimensions of the lobes are also age related; they are larger in younger women and undergo involution around and after menopause. Lobes in the medial quadrants of the breast develop later and undergo involution earlier than the lobes in the lateral quadrants [30]. During the malignant transformation of the structures of the sick lobe, new cancerous TDLUs and ducts [27] may develop and increase the dimension of the involved lobe. 

Young age strongly correlates with a high risk of local recurrence after breast-conserving surgery, whether or not radiotherapy is given [31]. This relationship is associated with the dimensions of the sick lobe, which is an important factor in determining the success of breast-conserving surgery.

### 4.2. Extent of the Disease: The Biological Timing of Malignant Transformation

The committed progenitor cells dispersed unevenly within the sick lobe may undergo malignant transformation under the influence of exogenous and endogenous oncogenic stimuli [15]. According to our hypothesis, the timing of this transformation is determined by the number of required genetic alterations, which are mostly acquired during the division of these cells. This hypothesis has been termed the hypothesis of biological timing. Malignant transformation may appear in a single locus within the sick lobe, more than one locus at the same time or with considerable time difference, or at a large number of loci, yielding the segmental, peripheral, and lobar patterns of malignant transformation, respectively, as discussed above.

The timing and the pattern of malignant transformation within the sick lobe are the main determinants of disease extent, in addition to the dimensions of the lobe. Malignant transformation may appear in a small segment of a large lobe, giving rise to a unifocal early breast cancer of limited extent (segmental pattern). Additional tumor foci may develop within the same lobe years or decades later and will be perceived as a local recurrence after the initial tumor has been excised. If the malignant transformation targets distant individual TDLUs (peripheral pattern), the process will be multifocal from its beginning. The extent of such malignancy will be determined by the dimensions of the sick lobe and the distance between the affected TDLUs; the disease may be extensive or of limited extent. Asynchronous involvement of additional TDLUs leads to local recurrence if the sick lobe was not completely removed by surgery. The lobar pattern of malignant transformation develops as a result of simultaneous alteration of the progenitor cells at many loci, and, in the extreme, the entirety of the sick lobe. Such tumors involve diffusely the larger ducts and many TDLUs within the sick lobe. These tumors are often extensive from the very beginning of their development (Figure 1). In one of our studies, diffuse in situ carcinomas had an average disease extent of 52.7 mm (range 16–180 mm) [32].

### 4.3. Extent of the Disease: Invasion beyond the Borders of the Sick Lobe

Invasion may appear at a single locus or (simultaneously or asynchronously) at several loci of the sick lobe involved by an in situ cancer. The invasive component may invade beyond the area of the sick lobe, especially in a more advanced stage of the disease. Two mechanisms may lead to the multifocality of the invasive component in breast carcinoma: separate invasive foci may develop independently from each other from in situ carcinoma in different parts of the sick lobe, or they may be a result of intramammary tumor spread via the (pre-)lymphatic system. The latter possibility may explain the influence of multifocality on the metastatic capacity of the tumors and on survival.

The rare diffuse invasive carcinomas develop simultaneously at many loci of the sick lobe and often invade without provoking any stromal reaction, which in other cases may limit the tumoral growth (Figure 2). The outcome is an extensive invasive process involving large parts of the breast and not confined to the area of the sick lobe. Most of these tumors are of the lobular type [33] and are large and extensive at the moment of their clinical or radiological detection. In one of our studies, these tumors had an average size of 55.9 mm (range 27–91 mm) [32].

### 4.4. Extent of the Disease: Cutoffs

There is no international consensus regarding the definition of extensive breast carcinoma. Two cutoffs, 15 mm and 40 mm, are used in the Van Nuys Prognostic Index scoring system [34], but this scoring system is limited to cases of ductal carcinoma in situ. Faverly et al. [5] defined extensive carcinoma as tumors having foci more than 1 cm apart, in contrast to breast carcinomas of limited extent, which were proposed by the authors as adequate candidates for breast-conserving surgery. Because the extent of the disease is defined as volume or area of the breast tissue including all the malignant structures within the breast, we prefer to use a cutoff defining the volume or the area and not the distance between the foci.

We define extensive tumors as those occupying a tissue area at least 40 mm in the largest dimension in contrast to breast carcinomas of limited extent [7, 9]. The most important reason for choosing this cutoff is the 10-year followup results regarding our material (1996–1998), presented in Tables 1, 2, and 3.

Testing different cutoffs (20 mm, 30 mm, and 40 mm) led to the conclusion that an extent of 40 mm or more represents the proper target cutoff in selecting cases for breast-conserving surgery. Such tumors comprised in our material one third of carcinomas in this series and exhibited a relative risk of 2.75 for developing ipsilateral local recurrence compared to nonextensive tumors if treated with conserving surgery and irradiation. Further, significant differences were seen in local ipsilateral recurrence rates when extensive tumors treated with mastectomy versus breast-conserving surgery were compared. Such statistically significant differences could not be demonstrated with 20-mm or 30-mm cutoff values. It is worth mentioning that an extent of the disease greater or equal to 40 mm also represents a survival-related negative prognostic parameter [24, 25].

### 4.5. Extent of the Disease: Relation to Tumor Size

We compared the extent of the disease and the distribution of the lesions in a consecutive series of 120 purely in situ carcinomas, 332 early invasive carcinomas (<15 mm), and 340 more advanced invasive carcinomas (≥15 mm) and found that the proportions of extensive cases in these categories were 45.0%, 42.5%, and 42.4%, respectively [9]. In another study on carcinomas 1–14 mm in size, we found that 96 of 301 (31.9%) had a multifocal invasive component and that none of them demonstrated a diffuse invasive growth pattern [8]. Thus, early breast carcinomas are as often extensive and as often multifocal as their more advanced counterparts; they differ from the advanced carcinomas in the smaller size of the individual invasive lesion(s).

### 4.6. Extent of the Disease: The Surrounding Normal Tissue

Genetic alterations similar or identical to those in cancer may be found in morphologically normal breast tissue, a finding strongly supporting the sick lobe hypothesis. Such alterations were demonstrated in normal-looking breast tissue as far as 4 cm from the cancer and even in breasts free of histologically verifiable cancer [14, 35]. Although the status of the surgical margins is clearly related to the risk of developing local recurrence, a clear margin, free of microscopic tumor foci, is not a guarantee that already developed distant tumor foci or a risk tissue carrying genetic abnormalities representing potential source of cancer foci have not been left behind after a seemingly complete intervention. Although postoperative irradiation substantially reduces the risk of local recurrence (Table 4), proper preoperative mapping of the disease and identifying the sick lobe are essential in planning adequate surgery. 

**Figure 4:**
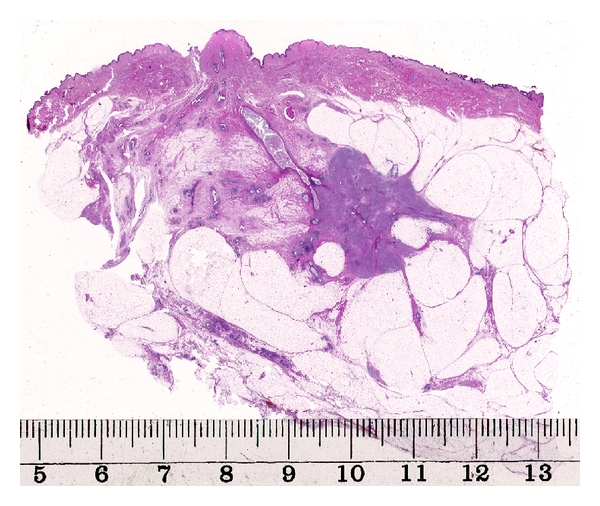
Invasive breast carcinoma with an in situ component involving a lactiferous duct leading to the invasive area.

The surgical intervention in early breast cancer must target excision of the already developed and radiologically and morphologically evident cancer foci together with the surrounding genetically altered but morphologically normal at-risk tissue. In other words, the aim is to remove the entire sick lobe together with the lesions within it; partial excision of the sick lobe represents a risk for tumor recurrence. Because of the above-discussed morphological variability, proper intravital mapping of the breast lobes and identifying the borders of the sick lobe is very difficult. Removing a lobe-like triangular piece of tissue from the breast (segmental excision) seems to be a more appropriate approach in context of the sick lobe theory than a simple lumpectomy. Modern breast ultrasound techniques may visualize the central axis of a lobe and lead the radiologist and the surgeon to excise the proper structures [30].  Figure 4 demonstrates a case of breast carcinoma with a duct leading into the area of the invasive tumor. Dooley propose routine operative breast endoscopy during lumpectomy to direct the surgical intervention towards the diseased part of the sick lobe [36]; his long experience with such an approach is reported in the present issue of the International Journal of Breast Cancer. 

Breast carcinomas of limited extent (occupying a tissue area <40 mm) are proper candidates for breast-conserving surgery. In other cases, careful individual preoperative assessment of disease extent is necessary in making decisions about the most appropriate surgical approach, taking into account the position of the lesion(s) within the breast, the dimensions of the breast, and patient preference.

## 5. Conclusions

Breast carcinoma is a lobar disease and, in the vast majority of cases, it is confined to the structures of a single sick lobe at its early stage. Finding the ductal tree of the sick lobe and mapping the diseased part(s) of it are essential in guiding adequate surgical intervention. Breast carcinomas of limited extent (<4 cm), whether unifocal or multifocal, are proper candidates for breast-conserving surgery. Adequacy of breast conservation in more extensive tumors should be carefully judged preoperatively in every individual case. In situ carcinomas with a lobar growth pattern (diffuse ductal carcinoma in situ) and invasive breast carcinomas of diffuse type often represent extensive disease, limiting the success of breast-conserving surgery.

## Figures and Tables

**Table 1:** Ipsilateral local recurrence rates by disease extent and type of surgery: extensive tumors defined as those occupying an area 4 cm or larger in the greatest dimension. Falun 1996–1998, 10-year followup.

	Extensive tumors ≥4 cm	Nonextensive tumors <4 cm	Total	Relative risk	Significance level
Mastectomy	7.3% (9/124)	9.3% (8/86)	8.1% (17/210)	RR = 0.7802 (CI: 0.3135–1.9429)	*P* = .5937
Breast-conserving surgery	20.5% (9/44)	7.4% (20/269)	8.9% (29/313)	RR = 2.7511 (CI: 1.3401–5.6478)	*P* = .0058
Sum	10.7% (18/168)	7.9% (28/355)	8.6% (46/523)	RR = 1.3584 (CI: 0.7736–2.3852)	*P* = .2862
Relative risk	RR = 2.8182 (CI: 1.1955–6.6435)	RR = 1.2512 (CI: 0.5717–2.7380)	RR = 0.8737 (CI: 0.4928–1.5490)		
Significance level	*P* = .0179	*P* = .5749	*P* = .6440		

**Table 2:** Ipsilateral local recurrence rates by disease extent and type of surgery: extensive tumors defined as those occupying an area 3 cm or larger in the greatest dimension. Falun 1996–1998, 10-year followup.

	Extensive tumors ≥3 cm	Nonextensive tumors <3 cm	Sum	Relative risk	Significance level
Mastectomy	7.5% (12/160)	10.0% (5/50)	8.1% (17/210)	RR = 0.7500 (CI: 0.2776–2.0261)	*P* = .5707
Breast-conserving surgery	15.0% (12/80)	7.3% (17/233)	9.2% (29/313)	RR = 2.0559 (CI: 1.0271–4.1152)	*P* = .0418
Sum	10.0% (24/240)	7.8% (22/283)	8.8% (46/523)	RR = 1.2864 (CI: 0.7404–2.2349)	*P* = .3716
Relative risk	RR = 2.0000 (CI: 0.9411–4.2502)	RR = 0.7296 (CI: 0.2824–1.8851)	RR = 1.1445 (CI: 0.6456–2.0291)		
Significance level	*P* = .0715	*P* = .5151	*P* = .6440		

**Table 3:** Ipsilateral local recurrence rates by disease extent and type of surgery: extensive tumors defined as those occupying an area 2 cm or larger in the greatest dimension. Falun 1996–1998, 10-year followup.

	Extensive tumors ≥2 cm	Nonextensive tumors <2 cm	Sum	Relative risk	Significance level
Mastectomy	7.7% (15/194)	12.5% (2/16)	8.1% (17/210)	RR = 0.6186 (CI: 0.1549–2.4699)	*P* = .4965
Breast-conserving surgery	12.0% (18/150)	6.7% (11/163)	9.3% (29/313)	RR = 1.7782 (CI: 0.8665–3.6407)	*P* = .1154
Sum	9.6% (33/344)	7.3% (13/179)	8.8% (46/523)	RR = 1.3209 (CI: 0.7135–2.4453)	*P* = .3758
Relative risk	RR = 1.5520 (CI: 0.8082–2.9766)	RR = 0.5399 (CI: 0.1310–2.2257)	RR = 1.1445 (CI: 0.6456–2.0291)		
Significance level	*P* = .1859	*P* = .3937	*P* = .6440		

**Table 4:** Ipsilateral local recurrence rates by disease extent and postoperative irradiation in extensive tumors and tumors of limited extent treated with breast-conserving surgery, Falun 1996–1998, 10-year followup.

Extent	Irradiated	Nonirradiated	Data missing	Sum	Relative risk	Significance level
≥4 cm	10.7% (3/28)	42.9% (6/14)	0.0% (0/2)	20.5% (9/44)	RR = 4.0000 (CI: 1.1709–13.6643)	*P* = .0270
<4 cm	3.9% (7/178)	15.2% (12/79)	50.0% (1/2)	7.4% (20/269)	RR = 3.8626 (CI: 1.5803–9.2208)	*P* = .0030
